# MicroRNA (miRNA) Signaling in the Human CNS in Sporadic Alzheimer’s Disease (AD)-Novel and Unique Pathological Features

**DOI:** 10.3390/ijms161226223

**Published:** 2015-12-17

**Authors:** Yuhai Zhao, Aileen I. Pogue, Walter J. Lukiw

**Affiliations:** 1 Louisiana State University Neuroscience Center, LSU Health Sciences Center, New Orleans, LA 70112, USA; yzhao4@lsuhsc.edu; 2 Department of Anatomy and Cell Biology, Louisiana State University Health Sciences Center, New Orleans, LA 70112, USA; 3 Alchem Biotek, Toronto, ON M5S 1A8, Canada; wlukiw@yahoo.com; 4 Department of Ophthalmology, LSU Neuroscience Center, Louisiana State University Health Sciences Center, New Orleans, LA 70112, USA; 5 Department of Neurology, LSU Neuroscience Center, Louisiana State University Health Sciences Center, New Orleans, LA 70112, USA

**Keywords:** Aβ42 peptide, age-related macular degeneration (AMD), amyloid clearance, heterogeneity of the disease process, inflammation, innate-immune response, microRNA (miRNA), miRNA-7, miRNA-9, miRNA-34a, miRNA-125b, miRNA-146a, miRNA-155, normal aging, prion disease, sporadic AD

## Abstract

Of the approximately ~2.65 × 10^3^ mature microRNAs (miRNAs) so far identified in *Homo sapiens*, only a surprisingly small but select subset—about 35–40—are highly abundant in the human central nervous system (CNS). This fact alone underscores the extremely high selection pressure for the human CNS to utilize only specific ribonucleotide sequences contained within these single-stranded non-coding RNAs (ncRNAs) for productive miRNA–mRNA interactions and the down-regulation of gene expression. In this article we will: (i) consolidate some of our still evolving ideas concerning the role of miRNAs in the CNS in normal aging and in health, and in sporadic Alzheimer’s disease (AD) and related forms of chronic neurodegeneration; and (ii) highlight certain aspects of the most current work in this research field, with particular emphasis on the findings from our lab of a small pathogenic family of six inducible, pro-inflammatory, NF-κB-regulated miRNAs including miRNA-7, miRNA-9, miRNA-34a, miRNA-125b, miRNA-146a and miRNA-155. This group of six CNS-abundant miRNAs significantly up-regulated in sporadic AD are emerging as what appear to be key mechanistic contributors to the sporadic AD process and can explain much of the neuropathology of this common, age-related inflammatory neurodegeneration of the human CNS.

## 1. Introduction

**Figure 1:**
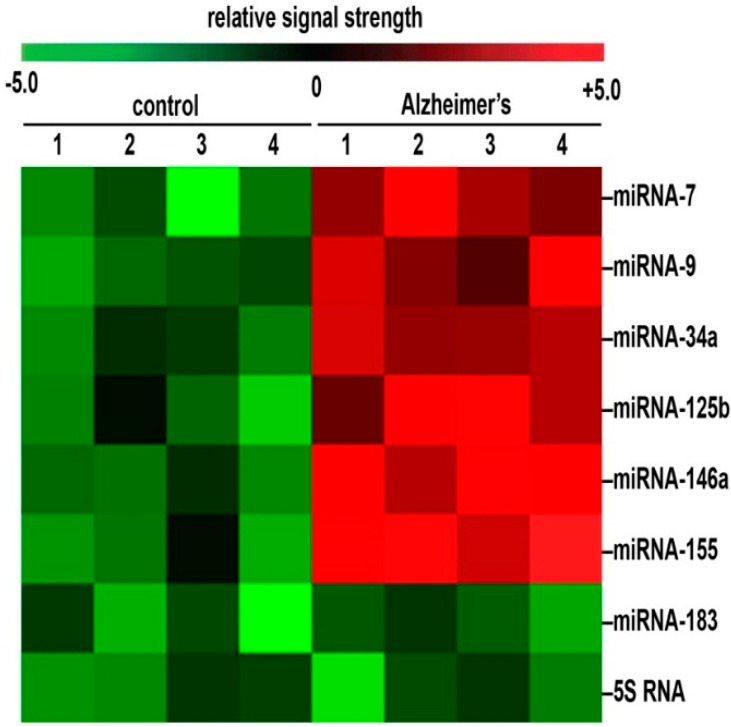
Representative heat map (“color-coded cluster diagram”) of the relative signal strength for six inducible, NF-κB regulated, pro-inflammatory, CNS-enriched miRNAs in four control and four sporadic Alzheimer’s disease (AD) brains all having post-mortem intervals (PMIs) of ≤2.1 h; the most significantly up-regulated miRNAs exhibited relative signal strengths that were miRNA-146a >>> miRNA-155 >> miRNA-125b >> miRNA-34a ≥ miRNA-9 ≥ miRNA-7; up-regulated miRNAs for this group ranged from 2.5-to-6-fold or more over age-matched non-AD controls; the control ncRNAs, including miRNA-183 and 5S RNA, were found not change significantly between age-matched controls and AD; in addition there were no significant differences in (**i**) age (control 71.5 ± 6.1 year; AD 72.2 ± 7.6 year); (**ii**) gender (all samples were from females); (**iii**) PMI (all samples <2.1 h); (**iv**) RNA quality (all samples **A_260/280_** ~2.1) and/or RNA integrity (RIN) values of >8.5 or higher or (**v**) total RNA yield (all samples had a mean yield of 1.25 μg RNA/mg wet weight brain tissue) between the control and AD brain samples; these six up-regulated miRNAs have a significant number of sporadic-AD-relevant mRNA targets that can explain much of the discernible neuropathology of the AD brain; (see text; and Figure 2); analogous miRNA profiles for the rarer forms of familial AD have not yet been analyzed to this extent.

**Figure 2:**
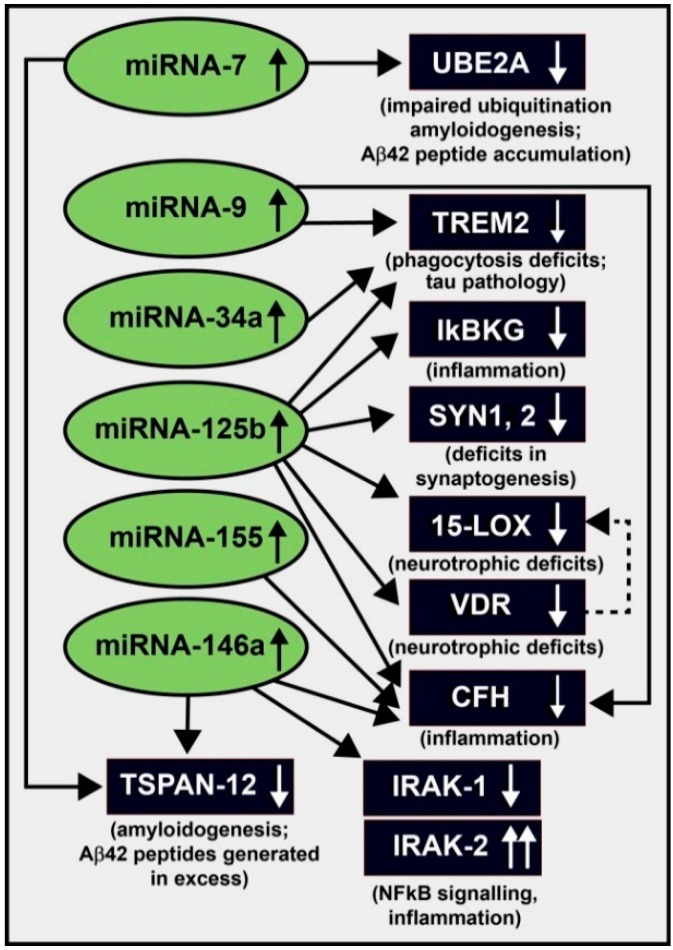
Highly schematicized interactive miRNA-mRNA signaling map of up-regulated miRNAs (green ovals) targeting mRNA 3′-UTRs (black squares) and down-regulating gene expression from these sporadic Alzheimer’s disease (AD)-relevant targets; as has been previously shown one miRNA can target multiple mRNA 3′-UTRs (*i.e.*, miRNA-125b) and conversely, multiple miRNAs can target a single mRNA 3′-UTR (*i.e.*, miRNA-155, miRNA-155 and CFH); this scheme is also reiterated (with references) in Table 1 (see above). Note that down-regulated regulatory mRNAs (such as the vitamin D receptor, VDR) may have ancillary effects on the transcriptional control of other RNA Pol II genes including 15-lipoxygenase (15-LOX; hatched line with arrow). Importantly this highly integrated gene expression signaling mechanism addresses the observed down-regulation in the expression of multiple mRNAs that are normally involved in physiological pathways that are known to be specifically targeted by the AD process; this miRNA-mRNA network is highly interactive and other miRNAs, mRNAs and miRNA-mRNA interactions may be involved. The inducible microRNAs miRNA-146a and miRNA-155 are typically found to be the most significantly increased miRNAs over age-matched controls (see Figure 1). Inhibition of the pro-inflammatory transcription factor NF-κB or full or partial blocking of the pathogenic induction of these six miRNAs using AM approaches may provide effective therapeutic benefit. However, the design of practical NF-κB or miRNA inhibition protocols, or the individual or combinatorial use of one or more inhibitory strategies, still remain open to very active pharmacological investigation [22,37,38,39,57,58,59,60,61,62,63]. Recent data using stressed human brain cells in primary culture has suggested that single or combinatorial pharmacological approaches may useful in the neutralization of these inducible, pathogenic gene expression programs to enable brain cells to re-establish homeostasis, and be of ultimate benefit in the therapeutic management of the AD process [37,38,39,63,64,65,66,67,68,69,70]. A family of at least 6 up-regulated microRNAs down-regulate the expression of at least 10 sporadic AD-relevant mRNAs and their expression, and can explain much of the neuropathology observed in the AD brain.

Currently predicted to regulate the expression of more than 50% of all protein-coding genes, microRNAs (miRNAs), ~21–25 nucleotide (nt) non-coding, single stranded RNAs (ssRNAs), have established themselves as important post-transcriptional regulators of messenger RNA (mRNA) abundance, complexity and speciation in the human central nervous system (CNS) in aging, development, health and disease [1,2,3,4,5,6,7,8,9,10,11,12,13,14,15,16]. Of the 2.65 × 10**^3^** different mature miRNAs in *Homo sapiens* so far identified, the CNS is highly represented by only a surprisingly small number of these miRNA species. In fact only about 35–40 miRNAs are abundant in the human brain, and this underscores the tremendous selection pressure carried by specific ribonucleotide sequences positioned within these non-coding RNAs (ncRNAs) [4,5,6,7,8,9,10]. Recent observations in the field of miRNA research specifically in the human brain has strengthened our previous concepts and have provided additional evidence that: (**i**) like messenger RNAs (mRNAs), the large majority of miRNAs are transcribed by a 550 kDa RNA polymerase II (RNA Pol II) into long precursor miRNAs (pre-miRNAs) which are subsequently processed into 21–25 nt mature, functional miRNA species [1,3,8,9,10,11]; (**ii**) mature miRNAs have short half-lives of about 1–3 h *in vivo* and are relatively unstable in the pro-degradative environment of AD tissues [12,13,14]; (**iii**) storage of miRNA in exosomes or other vesicular systems, adsorption onto RNA transport molecules, tertiary folding or other mechanisms may prolong miRNA biological half-life [14,15,16,17]; (**iv**) mature up-regulated miRNAs generally interact, via base pair complementarity, with the 3′-untranslated region (3′-UTR) of their target mRNA(s) to eliminate or down-regulate expression of that mRNA [8,9,10,18]; (**v**) one miRNA can target multiple mRNA 3′-UTRs and conversely multiple miRNAs can target a single mRNA 3′-UTR (Figure 1) [1,8,10,14,19]; (**vi**) up-regulated miRNAs for the most part down-regulate their mRNA targets via a “***productive***” 3′-UTR interaction [18,19,20]; (**vii**) miRNAs can act both as an “***on-off switch***” or as “***a fine-tuner***” of mRNA-driven gene expression via “***threshold effects***” [18,19,20,21]; (**viii**) miRNAs and their target mRNAs are more often than not transcribed from different chromosomes, so it may take gene transcription products from at least two different RNA Pol II transcribed chromosomes to orchestrate the expression of miRNA-regulated mRNAs [18,19,20,21,22]; (**ix**) certain miRNAs are highly conserved in sequence over evolution; for example miRNA-854 is conserved between *Arabidopsis* and *Homo sapiens* (organism divergence ~1.5 billion years); [23,24]; (**x**) only about one sixth of the 35–40 miRNAs that are abundant in the human CNS appear to be significantly elevated in expression in the AD brain and in stressed primary cultures of human brain cells (see below) [2,24,25,26,27]; (**xi**) the human brain appears to share certain common miRNAs with other neural ectoderm-derived tissues such as the retina [5,6,7,8,9,28,29,30,31,32]; (**xii**) chronic inflammatory neurodegenerative diseases of the retina such as age-related macular degeneration (AMD) as well as multiple sclerosis and prion disease also share an up-regulation of many of these same miRNAs [28,32,33,34,35,36]; (**xiii**) in stressed primary human brain cell co-culture experiments the same six miRNAs were shown to be up-regulated (as has been observed in in sporadic AD; miRNA-7, miRNA-9, miRNA-34a, miRNA-125b, miRNA-146a and miRNA-155), each were found to be inducible from outside the cell, and all six miRNAs were found to be transcriptionally regulated by the pro-inflammatory transcription factor NF-κB (see below) [33,37,38,39]; (**xiv**) these up-regulated miRNAs that characterize AD and chronic, progressive, inflammatory degeneration of the human brain are strongly associated with deficits in the homeostatic expression of brain gene families involved in amyloidogenesis, inflammation, neurotrophism, the innate-immune response, and synaptogenesis [33,37,38,39]; and (**xv**) many of these same six miRNAs can be induced by environmental and externally applied stressors such as aluminum and other metal sulfates, by neurotrophic viruses such as herpes simplex 1 (HSV-1) or by prion infection [34,35,36,40,41,42,43]. While other miRNAs and mRNAs circuits may be additionally involved, we find it intriguing that a select family of only six inducible miRNAs and their highly interactive reactions with multiple mRNA targets in the brain can explain many of the widely-recognized neuropathological characteristics of AD and other chronic, progressive inflammatory neurodegenerations of the human CNS (Figure 1 and Figure 2).

## 2. The High Selection Pressure and High Density of Genetic Information in Human Brain miRNAs

The integrated use of microfluidic chip-based miRNA arrays and next generation RNA sequencing technologies have recently identified ~2.65 × 10**^3^** different mature miRNAs in ***Homo sapiens***, and probably not many more novel, abundant, mature miRNA sequences are still to be discovered [4,5,6,7,8,9,10,11,12,13,14,15,16]. What is remarkable is the relatively small number of “***abundant functional microRNAs***” in the human CNS, and these currently number only about ~35–40 mature miRNA species [12,13,14,15,16,17,18]. One interesting mathematical and bioinformatics consideration is that a ~22 nt single-stranded ncRNA consisting of just four different ribonucleotides—(adenine, cytosine, guanine or uridine; A, C, G or U)—has the potential to generate in excess of 10**^13^** possible ~22 nt RNA sequence combinations. The observation that there are only about 2.65 × 10^3^ different miRNAs in the entire human organism, and only 35–40 significantly abundant miRNAs in the brain, suggests an unusually high selection pressure over evolution to employ only very specific miRNA oligonucleotide sequences that will function in neurobiologically useful miRNA–mRNA complementary interactions that ultimately regulate and shape global brain gene expression patterns [44,45,46,47,48,49,50,51,52]. Put another way, only about one in 10**^10^** potential miRNA sequences have found a useful purpose in miRNA-mRNA-based gene regulation in all of human biochemistry, neurochemistry and physiology, and less than one in 2 × 10**^11^** potential miRNA sequences have found a useful function in miRNA-mRNA-based gene regulation in transcriptome of the brain [3,4,5,6,7,8,9,48,49,50,51,52,53,54,55,56]. In addition, from what has recently been discovered, of the 35–40 significantly abundant miRNAs in the human brain only about 20%–25% are significantly up-regulated in the AD brain, and this has critical implications for the diagnostic and/or therapeutic strategies that may be useful for the detection and/or management of these pathologically up-regulated, inducible miRNAs in the degenerating CNS [11,12,13,14,15,16,17,18]. It remains a fundamental goal of future miRNA research to ascertain if these evolutionarily selected miRNA sequences in the brain represent some the common denominator of a complex miRNA-mRNA signaling scheme and/or are part of a cryptic neurological genetic signaling mechanism that we are just beginning to understand.

## 3. Up-Regulation of a Small Family of Six miRNAs Explains Many of the Neuropathological Deficits Seen in Sporadic Alzheimer’s Disease (AD)

As aforementioned the significant up-regulation of just six inducible miRNAs in sporadic AD and control brains and their targeting of selective mRNAs which are down-regulated can explain many of the neuropathological features that characterize the AD process (Figure 2). It is important to emphasize the overlapping and highly interactive pathological functions of multiple down-regulated mRNAs in AD-affected brain, and the sum of these pathological effects on global gene expression may well be much greater than the contribution of individual mRNA deficits. For example a miRNA-34a-mediated decrease in the expression of the phagocytic protein sensor and triggering receptor expressed in microglial/myeloid cells (TREM2) would not only result in defective phagocytosis and protein clearance pathways but the accumulating end-stage waste protein and peptides normally destined for degradation in the cytoplasm would also provide a basis for their accumulation, their self-aggregation into neurotoxic lesions resulting in a generalized increase in amyloidogenesis and an up-regulation of inflammation, all of which are highly characteristic of AD neuropathology (Figure 1, Table 1) [71,72,73,74,75,76]. Similarly, in both AD and in experimental model systems for AD an up-regulated miRNA-125b has also been found to down-regulate the expression of TREM2, IκBKG, SYN-2, 15-LOX, VDR and CFH that associate with deficits in phagocytosis, Aβ42 peptide clearance, NF-κB signaling, neurotrophism, deficits in the brain’s innate-immune response and an up-regulation of pro-inflammatory signaling (see below; Figure 2).

## 4. NF-κB Relevance and miRNA Signaling in AD

Keeping in line with the idea that AD involves a “***progressive, smoldering inflammation of the brain***”, several independent research laboratories have described specific increases in the abundance and/or activity of the transcription factor NF-κB in AD affected tissues [57,58,59,60,61,62,64,65,66,67,76]. This pro-inflammatory transcription factor comprises a small family of about ~20 heterodimeric DNA binding protein complexes; for example the relatively common ~115 kDa p50–p65 NF-κB factor, ubiquitous in brain tissues, normally resides as a pre-formed, quiescent transcription factor in the cytoplasm [62,64,65,66,67]. All six of the pro-inflammatory, inducible miRNAs described above—miRNA-7, miRNA-9, miRNA-34a, miRNA-125b, miRNA-146a and miRNA-155—are under transcriptional control by NF-κB (p50/p65) by virtue of multiple NF-κB binding sites in the regulatory regions of these six genes that yield miRNA precursors [32,64,65,66,67,68,69,70]. Cytoplasmic NF-κB is activated and NF-κB signaling is induced via a wide range of physiological stressors including Aβ40, Aβ42 and other ~40–42 amino acid amyloid peptides, bacterial infection, elevations in ambient reactive oxygen species (ROS), hypoxia, ionizing radiation, neurotoxic metals, pro-inflammatory chemokines and cytokines, viral infection, and other forms of physiological stress [63,66,67,68,69,70,77]. Interestingly most of these factors have been implicated in the initiation, propagation and/or neuropathology associated with AD [63,66,67,68,69,70,77]. NF-κB activation is largely mediated via the phosphorylation of serine residues located within a family of NF-κB inhibitory subunits, bound non-covalently to the NF-κB heterodimer, and known collectively as “***inhibitors of κB***” or “***IκB***”. IκB serine phosphorylation is subsequently followed by IκB degeneration via the cytoplasmic proteasome complex and through cooperation with NF-κB essential modulator (NEMO) proteins [65,66,67]. “***Activated NF-κB heterodimers***” are then able to translocate to the nucleus where they target binding sites on NF-κB-sensitive genes containing DNA sequences homologous to the canonical NF-κB-DNA recognition sequence 5′-GGGACTTTCC-3′ [66,67]. Ever since the original description of NF-κB almost 30 years ago several hundred natural and synthetic NF-κB inhibitors have been developed to inhibit NF-κB-activation and translocation within this complex signaling system [37,40,65]. Indeed, NF-κB inhibition can involve the blocking of NF-κB activation and/or signaling at various control nodes, from inhibition of the kinases that phosphorylate IκB serines (thus preventing NF-κB activation), to the deacetylation of the NF-κB p65 subunit, to the proteasome-mediated degradation of the IκB, to the translocation of NF-κB to sites within the nucleus, to the prevention of NF-κB recognition and binding to DNA sites using oligonucleotide decoy and/or antisense strategies [61,62,63,64,65,66,67,68,69,70,77]. Importantly, constitutive or “housekeeping” NF-κB activation and signaling is critical to multiple aspects of normal, homeostatic functions in the brain, and essential to the control of apoptosis, innate- and adaptive-immunity, cell proliferation, the inflammatory response and related neurophysiological stress responses. Hence NF-κB inhibition strategies may have unwanted and serious off-target effects. In addition, since NF-κB may have multiple off-target effects in different types of brain cells it will be important to critically understand specific anti-NF-κB inhibition protocols and approaches in different cell types of the CNS and other susceptible tissues under both control and pathological conditions in order to maximize beneficial pharmacological treatment outcomes [37,38,39,61,63,66,67,68,69,70,77].

With regards to this family of six inducible, NF-κB up-regulated miRNAs, these recent findings in part define a highly complex and interactive network of NF-κB-sensitive, up-regulated miRNAs in AD brain that explains much of the characteristic neuropathology associated with this neurological condition. As fore-mentioned, miRNA-125b is a highly expressed, CNS-abundant member of this up-regulated family of six miRNAs that may in part be responsible for driving deficits in Aβ42 peptide and related phagocytic clearance (via triggering receptor expressed in myeloid/microglial cells; TREM2), chronic innate-immune and inflammatory signaling (CFH, IκBKG), impairments in neurotransmitter packaging into vesicles and vesicular release (synapsin-2; SYN-2), and deficits in neurotrophism within the brain (15-lipoxygenase, 15-LOX; vitamin D receptor; VDR) (Table 1 and Figure 2). Similarly, other up-regulated, NF-κB-sensitive miRNAs (such as the strongly and rapidly induced miRNA-146a) may be responsible for the promotion of amyloidogenesis (tetraspanin 12; TSPAN12) and/or defects in NF-κB regulation (IRAK-1, IRAK-2) in AD-affected tissues; these inducible, up-regulated miRNAs that down-regulate their target mRNAs also appear to form a self-perpetuating and pathogenic miRNA-mRNA signaling network, due in part to chronic NF-κB re-activation perhaps through the involvement of recurring deficits in IκB signaling [60,61,62,63,64,65,66,67,68,69,70,77,78,79,80,81]. Inhibition of NF-κB activation and signaling and/or the individual quenching of the pathogenic up-regulation of these six miRNAs could provide novel therapeutic benefit for the clinical management of AD, however what specific NF-κB inhibition strategies or whether they can be utilized in some beneficial combinatorial approach remain open to very active investigation [57,58,59,60,61,62]. Recently published data using stressed primary human brain cells in culture has suggested that these pharmacological strategies may neutralize any or all of these chronic, age-related pro-inflammatory neurodegenerative gene expression programs to ultimately be of novel pharmacological benefit in the clinical management of AD and other progressive neuropathies of the human CNS [39,40,57,61,62].

## 5. NF-κB Inhibition *versus* Anti-miRNA (AM) Therapeutic Approaches

The relatively large number of physiological inducers of NF-κB signaling (see above) also up-regulate the transcription, expression and signaling of a specific family of NF-κB-sensitive pro-inflammatory miRNAs. These include, predominantly, miRNA-7, miRNA-9, miRNA-34a, miRNA-125b, miRNA-146a and miRNA-155 (Figure 1 and Figure 2). Because NF-κB can variably modulate gene expression (**i**) directly, via the transcriptional activation of NF-κB sensitive genes, *i.e.*, those containing single or multiple NF-κB binding sites in their promoters, with resulting increases in pro-inflammatory gene expression; and/or (**ii**) indirectly via an miRNA-mRNA 3′-UTR interaction and post-transcriptional down-regulation, it would be an important future goal to characterize in detail productive NF-κB-sensitive miRNA-mRNA signaling pathways to establish at what point anti-NF-κB or anti-miRNA (AM)-based therapeutic strategies may be best prescribed to fit the individual pathological situation [68]. While the up-regulation of NF-κB in part defines a pathogenic network of inducible, pro-inflammatory NF-κB-sensitive pre-miRNA genes, other pathologically-induced transcription factors, or combinations of transcription factors may define the induction of other families of pathogenic pre-miRNAs. One general rule to follow may be that a highly interactive network of NF-κB or other transcription factor-sensitive up-regulated miRNAs could define a pathological gene expression program involving ncRNAs that may be amenable to strategic manipulation at some point, to eventually normalize the neurobiological balance between inducible pro-inflammatory neurodegeneration or neurotrophic and brain cell survival signals. It should also be mentioned that recent miRNA array and RNA sequencing data and CSF analyses have further indicated that different human populations, or indeed even different individuals within those populations, who have sporadic AD may exhibit quantitatively different miRNA or other biomarker profiles associated with the disease, and this may require a more “***individualized***” or “***personalized***” treatment approach to obtain maximal efficacy in the clinical management of diverse populations of AD patients [68,78,79,80,81].

## 6. Summary

AD is the prime example of an insidious, age-related, uniquely human, ultimately fatal neurodegenerative disorder that is predominantly characterized by the progressive spatiotemporal erosion of cognitive function and memory. AD is accompanied by the initiation and chronic propagation of neuropathology originating in the limbic system that subsequently spreads into other anatomical regions of the brain’s association neocortex. The natural homeostatic abundance of brain-resident miRNAs are indicative of neurological health in the CNS, and miRNA translocation between brain cells and the CSF may be responsible in part for proliferation of the AD process [2,3,4,5,6,7,8,9,10,11]. Recently, independent neurological research laboratories have provided evidence for the up-regulation of a small family of six inducible, pathogenic miRNAs in AD and in several other age-related diseases involving a chronic and progressive inflammatory neurodegeneration such as multiple sclerosis, Parkinson’s disease and prion disease [33,34,35,36,71]. These six miRNAs include miRNA-7, miRNA-9, miRNA-34a, miRNA-125b, miRNA-146a and miRNA-155 (Figure 1 and Figure 2) [34,35,36,39,40,41,42,43,44,45,46,47]. Other miRNAs may be involved but are not as apparent as these six miRNAs using multiple miRNA array-based and RNA sequencing analysis of ~120 short post-mortem interval neurologically normal control and AD brains (data not shown; manuscript in preparation) [22]. Recently published research findings from the last year indicates that these six miRNAs more often than not share the following distinguishing features: (**i**) that all of these potentially pathogenic miRNAs are basally expressed in control human brain neocortex and hippocampus but are induced several-fold over their basal levels in AD [56,65,68,77]; (**ii**) that *in vitro* their expression may be triggered by a wide variety of environmental- and/or inflammation-linked physiological stressors, including pro-inflammatory cytokines and chemokines, amyloid beta (Aβ40, Aβ42) peptides , neurotoxic metal sulfates such as aluminum sulfate, neurotrophic viruses such as herpes simplex-1 (HSV-1) and prion infection [33,34,35,36,71]; (**iii**) that this family of six overexpressed pathogenic, pro-inflammatory miRNAs can be induced from outside of the cell in stressed human primary brain cell culture experiments [63,68,69,70,77]; (**iv**) that via the coordinate down-regulation of multiple mRNA targets (resulting in deficits in the homeostatic expression of genes encoded by target mRNAs) they regulate various pathophysiological factors characteristic of AD, including alterations in the innate-immune response, impairments in neurotrophism, deficits in NF-κB signaling, phagocytosis and the clearance of Aβ peptides, diminished synaptogenesis, and the chronic induction of progressive inflammation and amyloidogenesis (Figure 1) [23,24,25,26,27,28,29,30]; (**v**) that all six of these pro-inflammatory miRNAs are under transcriptional control by NF-κB (in most part by the p50/p65 heterotypic dimer) in human primary co-cultures of neuronal and glial cells and in anatomical regions of brain tissues affected by [22,23,24,25,26,27,28,29,30,59,60]; and (**vi**) that both NF-κB inhibition and anti-microRNA (AM) therapeutic strategies can effectively knock down miRNA-AD-associated expression in human primary brain cell culture experiments and in transgenic murine models of AD (TgAD), and may ultimately be of therapeutic use in the clinical management of AD [27,28,29,32,37,42,57,68,82].

## 7. Concluding Remarks

In summary, when compared to an unchanging 22 nt ***Homo sapien*** miRNA-183 and the ~120 nucleotide structural 5S ribosomal RNA (5S rRNA; 5SRNA) and other control markers within the same sample, an inducible, NF-κB-regulated six member miRNA family consisting of miRNA-7, miRNA-9, miRNA-34a, miRNA-125b, miRNA-146a and miRNA-155 are found to be amongst the most consistently up-regulated pro-inflammatory miRNAs found in AD brain when compared to age-matched controls (Table 1; Figure 1 and Figure 2). This family of six pathogenic miRNAs form a highly cooperative miRNA-mRNA signaling network that can in part explain the down-regulation of selective AD-relevant brain genes involved in amyloidogenesis, phagocytosis, the innate-immune response, synaptogenesis, neurotrophism and NF-κB signaling that also collectively support chronic inflammatory neurodegeneration (Table 1; Figure 1 and Figure 2). Even this relatively small number of ~6 miRNAs and ~10 mRNAs involved, out of a total number of ~2.65 × 10^3^ miRNAs and ~2.5 × 10^4^ mRNAs in a typical human cell, creates an extremely complex, multicomponent neural signaling network (Figure 1 and Figure 2) [51,52,53,54,55,56,68,81]. Indeed this high degree of regulatory sophistication has likely introduced functional vulnerabilities into this network, which overall, predisposes the human CNS to high potential for age-related signaling malfunctions that can initiate and drive the sporadic AD process and other related forms of chronic, inflammatory neurodegeneration.

**Table 1:** Consequences of Up-regulated microRNAs (miRNAs) and Down-regulated messenger RNAs (mRNAs).

miRNA (up-Regulated)	Target mRNA (down-Regulated)	Energy of Association E_A_ (kcal/mol)	Consequence of Interaction (down-Regulated mRNA)	References
miRNA-7	UBE2A, TSPAN-12	−2.2 to −22.9	amyloidogenesis	[32,81]
miRNA-9	CFH, TREM2	−22.2	inflammation, innate-immune signaling, phagocytosis	[22,32,70,71,72,73,74,75,77]
miRNA-34a	TREM2	−25.2	phagocytosis; Aβ42 peptide clearance	[22,32,64,71,72,73,74,75,81]
miRNA-125b	15-LOX, CFH, IκBKG, SYN-2, TREM2, VDR	−21.7 to −29.5	inflammation, neurotrophism, phagocytosis, synaptogenesis	[22,32,64,68,71,72,73,74,75,81]
miRNA-146a	CFH, IRAK-1, TSPAN-12	−24.5 to −26.4	amyloidogenesis, inflammation, innate-immune signaling, NF-κB signaling	[32,39,63,64,81]
miRNA-155	CFH	−26.1	inflammation, innate-immune signaling	[32,33,39,64,81]
